# Impact analysis of cooperative perception on the performance of automated driving in unsignalized roundabouts

**DOI:** 10.3389/frobt.2023.1164950

**Published:** 2023-08-15

**Authors:** Hazim Zainudin, Konstantinos Koufos, Graham Lee, Lintong Jiang, Mehrdad Dianati

**Affiliations:** Warwick Manufacturing Group (WMG) at The University of Warwick, Coventry, United Kingdom

**Keywords:** connected and automated vehicles, cooperative perception, mobile edge computing, motion planning, surrogate safety assessment model, vehicle-to-everything communication

## Abstract

This paper reports the implementation and results of a simulation-based analysis of the impact of cloud/edge-enabled cooperative perception on the performance of automated driving in unsignalized roundabouts. This is achieved by comparing the performance of automated driving assisted by cooperative perception to that of a baseline system, where the automated vehicle relies only on its onboard sensing and perception for motion planning and control. The paper first provides the descriptions of the implemented simulation model, which integrates the SUMO road traffic generator and CARLA simulator. This includes descriptions of both the baseline and cooperative perception-assisted automated driving systems. We then define a set of relevant key performance indicators for traffic efficiency, safety, and ride comfort, as well as simulation scenarios to collect relevant data for our analysis. This is followed by the description of simulation scenarios, presentation of the results, and discussions of the insights learned from the results.

## 1 Introduction

Automated driving systems (ADSs) are expected to transform the transport of goods and people in terms of the safety of road users, the comfort of the operators and passengers, the efficiency of transport systems in terms of their carbon footprint, and the affordable accessibility of mobility services for the public^1^. To reap such benefits, solid evidence shall be provided to ensure that ADSs outperform human-driven vehicles to gain consumers’ trust in automated driving technologies (Boualam et al., 2022). This requires that ADSs can faithfully perceive the environment and the intentions of other road users, carry out accurate localization and mapping, plan safe motions, and execute robust control of the vehicle.

Despite recent progress in sensing capabilities, 3D object detection, and tracking, the ADS based on sole reliance on onboard sensing and perception has well-known limitations (Arnold et al., 2022). An alternative promising approach is to enhance onboard sensing through sensor data collected from multiple spatially diverse viewpoints, also known as cooperative perception. A powerful variant of cooperative perception is to deploy roadside infrastructures near junctions with object detection and edge computing capabilities that can collect information (raw sensor data or semantic perception information) from various sources, fuse them together, and broadcast the combined information over vehicle-to-everything (V2X) communication technologies to the surrounding vehicles. Therefore, connected and automated vehicles (CAVs) can combine their local understanding of the environment with the information received over the air from the infrastructure for improved decision-making and control.

Jandial et al. (2020) introduced an intelligent roadside unit (RSU) that processed sensor data from infrastructure cameras and lidars. RSUs were deployed at several target locations to support CAVs and were found to be particularly efficient at roundabouts and S-shaped roads, where the onboard perception is inadequate due to restricted field of vision and obstructions. Vijay et al. (2021) proposed a novel method for determining the optimal positioning of roadside sensors to maximize their coverage. They used a sensor model based on ray casting for correctly estimating the line-of-sight coverage, which leads to optimal sensor placement even under worst-case scenarios such as multiple 3D obstructions. If an adequate number of suitable sensor locations is identified, the proposed framework can prove extremely useful for infrastructure sensing. Motivated by the promises of cooperative perception, this study focuses on the comprehensive analysis of its impacts on automated driving in unsignalized roundabouts, as it has been found that unsignalized roundabouts result in shorter travel times and fewer accidents than traditional intersections with traffic lights or stop-signal control (Retting et al., 2001).

### 1.1 State-of-the-art review

Considering the roundabout as a shared space, conflicts between vehicles must be resolved. There are two main approaches to tackle the challenges between CAVs involved in a conflict. The vehicles can handover their control to a *road traffic manager*, which an entity that manages the flow of traffic (Zhao et al., 2018; Danesh et al., 2021; Martin-Gasulla and Elefteriadou, 2021; Wu and Zhu, 2021), or the vehicles can directly communicate among each other and negotiate the right of way (Debada and Gillet, 2018; Hang et al., 2021). The former approaches are known as centralized and can be further subdivided into reservation-based and trajectory-based optimization methods, with the latter gaining momentum due to recent advances in edge computing (Yu et al., 2019). The key assumption in centralized control is that all CAVs must comply with the trajectories calculated by the *road traffic manager*.

Wu and Zhu (2021) compared signalized intersections with signal-free roundabouts in pure CAV environments with central coordination and highlighted that for low-to-medium road traffic densities, the average waiting time at the entrance of roundabouts is less. Danesh et al. (2021) designed a system where the *road traffic manager* controls the arrival time for CAVs at the entrance of the roundabout to ensure sufficiently large gaps for safety and optimizes their trajectories within the roundabout to maximize passenger comfort. Furthermore, several priority resolution strategies were simulated in Martin-Gasulla and Elefteriadou (2021), with the best strategy increasing the roundabout capacity by 73% as compared to human driving at the cost of computational complexity in the *road traffic manager* strategy. Model predictive control (MPC) has also been used for traffic flow coordination of autonomous vehicles (Cao and Zoldy, 2021). The goal of MPC is to optimize the predictions of future states of the vehicles by iteratively processing inputs over a limited time horizon. A *road traffic manager* design for controlling the vehicle’s trajectory through a roundabout was presented in Cao and Zoldy (2021), where the performance of the MPC-based controller was evaluated with respect to the target speed.

In decentralized approaches, it is difficult to develop a generic method that allows CAVs with their own set of dynamics, sensors, communication devices, and characteristics to work together effectively. Negotiations between vehicles can use either the noncooperative game theory, such as the Stackelberg games in Hang et al. (2021), or cooperative game theory, such as forming coalitions in Hang et al. (2021) and the prisoner’s dilemma in Banjanovic-Mehmedovic et al. (2016). In the game theory framework, different vehicles are essentially independent players who can adapt their payoffs to reflect personalized driving behaviors, e.g., a conservative driver cares more about safety than driving efficiency (Hang et al., 2021). If the CAVs exchange only their current states, e.g., location and speed, every vehicle must also predict the trajectory of other vehicles to identify feasible merging gaps (Debada and Gillet, 2018). The study in Hang et al. (2022) showed that CAVs the ability to mimic cautious, average, and aggressive driving styles. It was shown that including sophisticated motion prediction of other vehicles in the decision-making process improved safety. Finally, the study in Wang et al. (2022) suggested a framework for the interaction-aware evaluation of the CAV’s safety while entering a roundabout. The study employed social value orientation and level-k game theory to characterize the interactive behaviors.

It is also worth noting that the transitional phase from manually driven to autonomous vehicles has been forecasted to take several decades, and mixed-traffic scenarios, where conventional vehicles are also present, will be inevitable (Bansal and Kockelman, 2017). In the mixed-traffic phase, conventional vehicles are likely to induce a negative effect on travel time for CAVs because human drivers tend to slow down near the entrance of roundabouts, unnecessarily sometimes, resulting in the formation of queues (Zhao et al., 2018). We may therefore need a high penetration of CAVs before witnessing noticeable gains in travel times and road traffic throughput of roundabouts.

### 1.2 Contributions

Instead of considering the roundabout as a system, we examine, in this paper, a single CAV, hereafter referred to as the EGO vehicle, which travels across a single-lane roundabout with four arms. All other vehicles are manually driven; hence, a low penetration of CAVs, which will be typical in the next few years, is essentially modeled. A key component in our system architecture is a fixed RSU equipped with a processing unit and wireless connectivity, which enhances the situation awareness of the EGO vehicle, but it does not influence its decision-making ability. Furthermore, it is reasonable to expect that in the coming years, several vehicles will carry communication units to assist human drivers. In this paper, we assume that all vehicles near a roundabout can communicate their intended paths to the RSU over a V2X air interface. The RSU can combine the information received over the air from the surrounding vehicles and create a single collective perception message (CPM) that is periodically broadcast using V2X. As a result, the EGO vehicle can finally combine its local perception with the additional information provided by the RSU for improved decision-making. The main contributions of this article are summarized as follows.

• We define a set of key performance indicators (KPIs) for traffic efficiency, safety, and ride comfort that are relevant for driving across urban unsignalized roundabouts.
• We build a co-simulation environment integrating SUMO and CARLA, where the former is used to generate random urban road traffic and the latter is used to implement motion planning algorithms.
• The key takeaway of the simulation experiments conducted in this article is that an EGO vehicle leveraging cooperative perception can traverse the roundabout quicker than an EGO vehicle that relies only on its onboard sensors to perform that. At the same time, cooperative perception helps improve the comfort of passengers during the maneuver without compromising safety.
• Even though this study focuses on roundabouts, we believe that the adopted methodology for quantifying the benefits of RSUs will be of interest to road authorities for the traffic management of CAVs near any other complex junction.

The tradeoff between comfort and travel time for an autonomous vehicle has been recently investigated in Zheng et al. (2021) with the goal of avoiding overly conservative maneuvers while driving across a roundabout. The motion planning module selects the trajectory that minimizes the weighted sum of travel time and discomfort, with the latter expressed by the integral of squared horizontal acceleration during the maneuver. Unfortunately, the constraints imposed by other traffic objects on the autonomous vehicle are not taken into account in the evaluation of the tradeoff in Zheng et al. (2021), as we will do in this paper.

### 1.3 Overview of the contents of the paper

The rest of this paper is organized as follows. The high-level system model architecture is presented in Section 2. Section 3 and Section 4 present the details of the simulated motion planning algorithm using only onboard perception and cooperative perception, respectively. The KPIs for driving efficiency, passenger comfort, and safety are explained in Section 5. The co-simulation environment between CARLA and SUMO is discussed in Section 6. Finally, Section 7 assesses the benefits of cooperative perception, and Section 8 concludes this study.

## 2 System model and high-level system architecture

**FIGURE 1:**
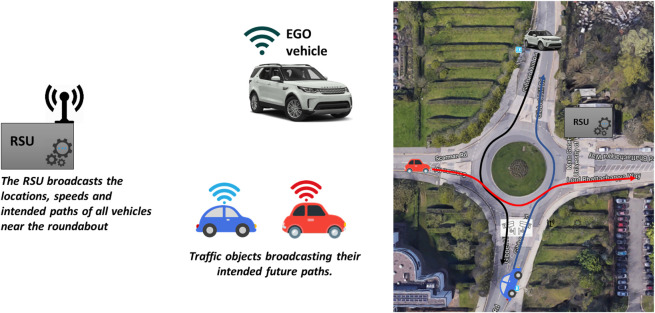
Google Maps view of the roundabout connecting the Gibbet Hill Road with the Lord Bhattacharyya Way at the University of Warwick overlaying the location of the RSU and the high-level system architecture. Example routes for the EGO vehicle and two traffic objects are also illustrated.

We consider an EGO vehicle mounted with perception sensors, such as cameras and lidars, and an onboard communication unit (OBU) that implements a V2X radio air interface. The EGO vehicle approaches the roundabout from the north entrance, see Figure 1, but due to occlusions, the onboard sensors can miss vehicles approaching the roundabout from another direction. To assist the EGO vehicle in traversing the roundabout efficiently and safely, an RSU empowered with a processing unit and V2X communication technology can be deployed near the junction. The RSU receives maneuver coordination messages (MCMs) (ETSI, 2022-10) from surrounding CAVs over ITS-G5, combines the received information, and packs it in the form of CPMs that are broadcast every 100 ms (ETSI, Release 2, 2019-12). The OBU on the EGO vehicle decodes the message broadcast and leverages the additional information for improved decision-making and motion planning.

In this paper, we conduct a simulation study to gauge the impact of edge computing and V2X communication on the driving performance of the EGO vehicle. Specifically, we compare the driving performance between the following two systems: in the former system, the EGO vehicle solely relies on onboard sensing, and in the latter system, the EGO vehicle’s perception is enhanced with additional information provided by the infrastructure. This comparison would be of interest to road authorities to decide whether it is cost-effective to install an RSU near the roundabout.

## 3 Baseline system: onboard alone perception

**FIGURE 2:**
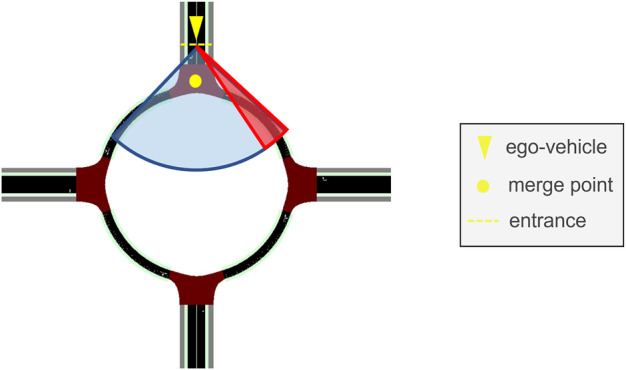
Field of vision (blue-shaded area) and area of concern (red-shaded area) for the baseline algorithm. The EGO vehicle, the merge point, and the entrance at the north leg are also illustrated.

In this section, we describe the system where the EGO vehicle relies only on onboard sensing for decision-making and motion planning without being connected to the infrastructure. It is assumed that the onboard sensor’s field of view is a cone with a range of 50 m and an azimuth of 90^*o*^ degrees; see the blue-shaded area in Figure 2. The selected sensor range value is quite conservative considering the range of modern lidar and camera sensors; hence, it is natural to assume that the object detection performance is ideal, meaning that the EGO vehicle can detect other road users in an error-free manner within the field of view of its sensors. The output of the onboard perception module is continuously sent into the motion planning module of the EGO vehicle, which is hereafter referred to as the baseline driving algorithm.

### 3.1 Baseline driving algorithm

The baseline algorithm replicates the decision-making process using only onboard perception. Decision-making starts at the north entrance, where the EGO vehicle has to decide when to enter the roundabout. The red-shaded area, partially overlapping with the blue-shaded area in Figure 2, is the area of concern during decision-making. This is the typical area that a human driver checks to the side at the entrance. The urban roundabout depicted in Figure 1 has an approximate radius equal to 15 m, which means that for the selected sensor azimuth and range values, the EGO vehicle, once it arrives at the entrance, can detect all objects within the upper quadrant of the roundabout, including the area of concern.

As the EGO vehicle approaches the entrance, it must slow down and assess whether it is safe to proceed by checking the area of concern. To do that, its target speed is first set at a low value, e.g., *V*_*T*_ = 2.2 m/s (or 5 mph). If there are vehicles within the area of concern, the target speed is reset at *V*_*T*_ = 0, which means that the EGO vehicle has to stop and wait at the entrance until the area of concern becomes completely clear. If the red-shaded area is clear to begin with, a new higher target speed is set, and the EGO vehicle enters the roundabout. The described decision-making process is conservative because there is not always a need to stop when merging into the ring, even if vehicles are in the area of concern. Even if these vehicles do not exit to the north, the EGO vehicle may go ahead, provided that the gap is large enough and the relative speed is low. Nevertheless, the baseline algorithm forces the EGO vehicle to stop when another vehicle is within the red-shaded area because it is natural to expect that CAVs should adopt cautious behavior planning in the presence of manually driven vehicles. For the same reason, the EGO vehicle does not rely on detecting the blinkers of other vehicles in the area of concern. In addition, the size of the selected roundabout in Figure 1 is small, with a radius of approximately 15 m, and has a single lane, which further justifies the adoption of conservative decision-making for the baseline algorithm.

The baseline algorithm modulates the EGO vehicle’s speed by setting the target speed *V*_*T*_ and controlling its instantaneous speed using a PID controller. Note that a PID controller is readily available in CARLA, but some adjustments had to be made. Specifically, to determine the target speed, the baseline algorithm uses the speed limit *v* *_max_*, the speed of the leading vehicle *v*_*l*_ (if any), and the road curvature. The algorithm first calculates the time-to-collision (TTC) to the leading vehicle, and if this is less than 3 seconds, it sets the target speed equal to the speed of the leading vehicle, i.e., *V*_*T*_(*t*) = *v*_*l*_(*t*), where *t* is the current time step. Otherwise, the target speed is set equal to the speed limit *V*_*T*_(*t*) = *v* *_max_*. As the driver response time ranges from 0.7–1.5 s, depending on the scenario and driver, it is safe to assume a safety factor of 2 using the upper bound of this range, i.e., 1.5 s (Drozdziel et al., 2020). This explains the selection of the cut-off threshold for TTC in setting the target speed of the EGO vehicle. While the TTC threshold should depend on the driving speed and, in general, on the driving scenario/environment, we have used just a single value (TTC = 3 s) because the only scenario simulated in this paper is an urban roundabout with a speed limit of 13.4 m/s (or 30 mph).

Finally, the target speed has to be adjusted using the road curvature for the purpose of ride comfort. The more curved a road is, a larger steering angle will be forced, which will increase the lateral acceleration assuming a constant speed throughout the curve. This might not be feasible for both driver/passenger comfort and steering characteristics. To maintain a comfortable level of lateral acceleration, the speed of the EGO vehicle around corners is lowered. Given the initial target speed of the EGO vehicle, $V_{T_{\mathrm{init}}}$ , and the input steering angle, *θ*, the adjusted target speed *V*_*T*_ can be expressed as

$$V_{T}=V_{T_{\min}}+\frac{\theta }{\theta_{\max}}\sigma_{f}V_{T_{\mathrm{init}}},$$ (1)

where $V_{T_{\min}}$ is the minimum target speed of the EGO vehicle (without a stopping decision), *σ*_*f*_ is a factor to tweak the sensitivity of the speed adjustment, and *θ*_*max*_ is the maximum input steering angle.

**FIGURE 3:**
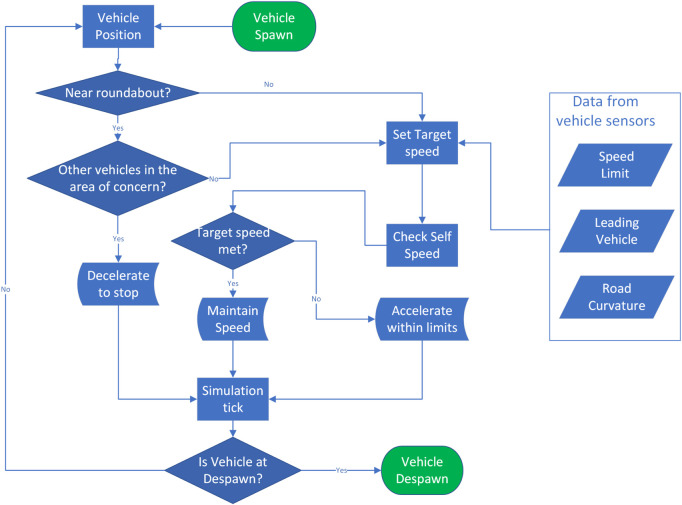
Block diagram for the baseline algorithm. All decision-making steps are repeated every 50 ms.

Before continuing with the description of the system leveraging cooperative perception, we summarize the decision-making steps of the baseline algorithm in a block diagram in Figure 3.

## 4 Cooperative perception-assisted system

In this section, we describe the system where the EGO vehicle leverages cooperative perception for motion planning. Targeting a future mobility scenario, it is assumed that all vehicles are connected (but not yet automated) and share their intended paths with the RSU. Even though the maneuver coordination message (MCM) is in the early stages of standardization, vehicles will likely exchange their intentions with other vehicles or the infrastructure near complex junctions (ETSI, 2022-10). Since the roundabout considered in this paper only has a single lane, it is sufficient that each vehicle indicates to the RSU which exit it will take. The RSU collects the MCMs transmitted by all vehicles and combines them into a single message, which contains the instantaneous locations, speeds, and intended paths of all vehicles. The message is broadcast every 100 ms, similar to the CPM. All communication between traffic objects, the EGO vehicle, and the RSU is assumed to be ideal, meaning that the received information is error- and delay-free, and there is no noise or loss during transmission and reception. In practice, the communication unit of the RSU must have good line-of-sight connectivity to all arms of the roundabout, e.g., being mounted on a pole at the center of the roundabout island. Evaluating the effects of imperfect V2X communication on cooperative perception requires future work, but this will not alter the performance evaluation methodology. The EGO vehicle exploits the information provided by the RSU for enhanced motion planning, which is hereafter referred to as the enhanced driving algorithm.

### 4.1 Cooperative perception-assisted automated driving algorithm

**FIGURE 4:**
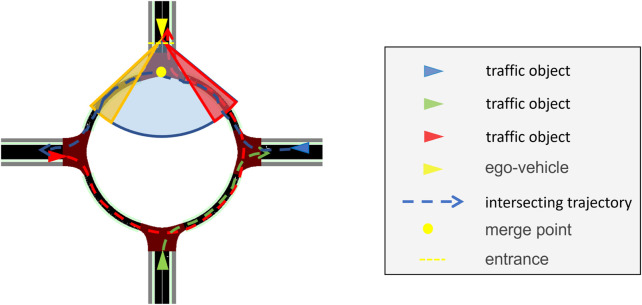
Enhanced algorithm field of vision (blue-shaded area) and area of concern (red- and amber-shaded areas). The red, green, and blue arrowed lines depict the intended paths of the similarly colored vehicles, respectively. The blue vehicle is a hazard if it is predicted to be within the amber-shaded area simultaneously with the EGO vehicle.

**FIGURE 5:**
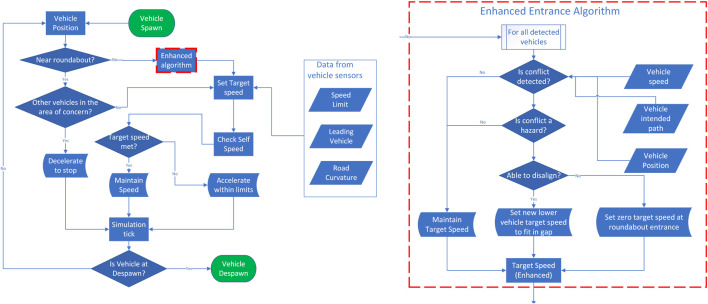
Block diagram for the cooperative perception-assisted automated driving algorithm. All decision-making steps are repeated every 50 ms. As compared to the baseline driving algorithm described in Figure 3, only the ‘enhanced algorithm’ block with the dashed-red outline has been added, which is detailed on the right-hand side and is executed every 100 ms.

The EGO vehicle processes the information received by the RSU and anticipates whether other vehicles in and around the roundabout are hazards. A hazard, in this case, is a vehicle that has a path intersecting with the desired path of the EGO vehicle; see the amber-shaded area in Figure 4, while also reaching that area when the EGO vehicle is also there. To identify whether a vehicle with an intersecting path is actually a hazard, the enhanced algorithm applies a rudimentary prediction model assuming that the traffic object maintains its velocity at the time of calculation. High-definition maps, using, for instance, curvilinear coordinates as in Masi (2021), can be used to calculate the traveled distance of other vehicles and, therefore, determine whether a vehicle is a hazard to the EGO vehicle or not. Upon detecting a hazard, the EGO vehicle tries to disalign itself from a potential collision. For safety reasons, it is assumed that the EGO vehicle can only decelerate as it approaches the north entrance of the roundabout. If the enhanced algorithm is unable to disalign itself from a potential collision, the EGO vehicle decelerates to stop, and the red-shaded area comes into play, functioning exactly like in the baseline algorithm. The decision-making steps of the enhanced automated driving algorithm are illustrated in Figure 5.

As compared to the baseline driving algorithm, a potential time save could be the case when there is a traffic object within the red-shaded area given in Figure 4, but its path does not intersect with that of the EGO vehicle. In this case, the baseline algorithm would have kept the EGO vehicle static till the red area becomes clear, while the enhanced algorithm triggers the EGO vehicle to enter the roundabout, as the traffic object is not a hazard despite being within the red-shaded area. For safety purposes, it is assumed that the baseline driving algorithm does not rely on detecting the blinkers of vehicles within the red-shaded area.

## 5 Key performance indicators

To fully assess the effectiveness of the simulated systems, several key performance indicators (KPIs) are used, such as driving efficiency, ride quality, and safety.

### 5.1 Driving efficiency metrics for the EGO vehicle

Driving efficiency focuses on how quickly the EGO vehicle can finish its maneuver and all factors that affect it. This can, for instance, be captured by the journey completion time and the stationary waiting time at the entrance of the roundabout.

#### 5.1.1 Journey completion time

The journey completion time is the required time for the EGO vehicle to drive through the designated route and finish at a specific point at the exit of the roundabout, while satisfying specific constraints on ride quality and safety.

#### 5.1.2 Stationary waiting time

A secondary metric that is related to the journey completion time is the stationary waiting time at the entrance of the roundabout. This measures the time the EGO vehicle remains stationary before entering the roundabout while waiting for other traffic objects to clear. It is expected that infrastructure-based sensing improves the EGO vehicle’s perception and awareness of its surroundings. This, in turn, can reduce the stationary waiting time and enhance driving efficiency. For instance, the EGO vehicle can become aware of traffic objects and their intended trajectories in advance. Provided that the desired trajectory of the EGO vehicle does not intersect with the intended trajectories of other traffic objects, the EGO vehicle can immediately enter the roundabout. On the contrary, without the infrastructure, the EGO vehicle must slow down and cautiously check for other traffic objects in its vicinity. Therefore, the infrastructure can potentially enhance traffic efficiency without compromising on safety and ride quality.

### 5.2 Ride quality metrics for the EGO vehicle

The most widely used ride quality measures are the instantaneous acceleration *a*(*t*) and jerk $k\left(t\right)=\frac{da}{dt}$ at a specific point in time *t*. The literature indicates that an absolute jerk of less than 1 m/s^3^ and an absolute acceleration of less than 2 m/s^2^ are associated with comfortable driving (Bae et al., 2019). The time granularity to calculate instantaneous KPIs would be equal to the time granularity of the simulator, which is 50 ms in our case. In addition, we use a two-dimensional model for the roundabout; hence, the momentary (or instantaneous) acceleration and jerk will be calculated only at the horizontal plane. Ride comfort can also be assessed using a sliding window averaging the acceleration over intervals of 5 s, but due to the lack of space, the associated results are not presented. Finally, it shall be noted that motion sickness is usually evaluated in the order of minutes; hence, KPIs associated with it are not relevant for the subject maneuver.

### 5.3 Safety metrics

Most existing approaches evaluating road traffic safety tend to lean toward metrics calculating vehicle proximity and speed. Hence, the safety metrics that best match our simulation scenario are the time-to-collision (TTC) and the post-encroachment time (PET) between the EGO vehicle and other traffic objects. The PET resembles the time headway between vehicles but is more tailored to the roundabout scenario, as we will shortly explain (Cooper, 1984).

#### 5.3.1 Time-to-collision

The TTC is mainly used to assess safety in adaptive cruise control systems. It is equal to the time the EGO vehicle has before colliding with another vehicle under the assumption that both vehicles maintain their trajectories at the moment of calculation. In its simplest form, the TTC assumes that the vehicles involved in the conflict maintain constant speeds equal to those at the moment of calculation.

$$\text{TTC}=\frac{h_{d}}{v_{f}-v_{l}},\;\text{subject to:}\;v_{f}>v_{l},$$ (2)

where *v*_*f*_ is the speed of the following vehicle, *v*_*l*_ is the speed of the leading vehicle, and *h*_*d*_ is their headway distance at the moment of calculation.

This metric is valid only if the EGO vehicle has an instantaneous speed higher/lower than that of its leading/following vehicle at the moment of calculation, as this is the only situation where a collision is imminent. More advanced TTC evaluation algorithms may also involve the instantaneous accelerations.

#### 5.3.2 Post-encroachment time

**FIGURE 6:**
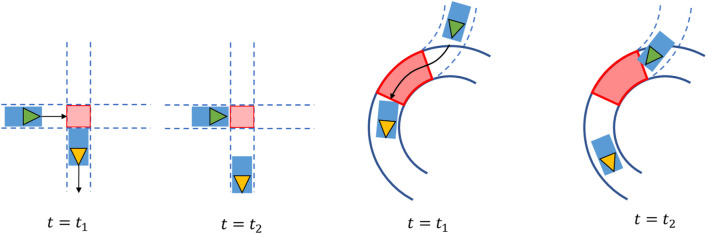
Post-encroachment time for T-junction (left) and roundabout (right) crossings.

To measure safety, the PET focuses on the overlap in the trajectories of the vehicles involved in the conflict. The PET is the time elapsed between the point where the leading vehicle leaves the intersection area, see the red-colored area in Figure 6, and the following vehicle enters that area.

In Figure 6, the green and yellow vehicles are the following and leading vehicles, respectively. The arrows depict their direction of travel. At *t* = *t*_1_, the leading vehicle has just left the red-shaded area, and at *t* = *t*_2_, the following vehicle is just about to enter that area. Therefore, the PET is equal to PET = *t*_2_ − *t*_1_.

## 6 Simulation model

In this section, we present our simulation setup starting from the creation of the map of the area and continuing with the control of the EGO vehicle in CARLA^2^ and the generation of random road traffic in SUMO. The EGO vehicle used in CARLA is the Tesla model 3^3^, which corresponds to a regular-size sedan vehicle type. The normal highway code for driving across roundabouts has been implemented in CARLA and SUMO, e.g., vehicles entering the roundabout will yield to vehicles already in the roundabout.

### 6.1 Map creation

**FIGURE 7:**
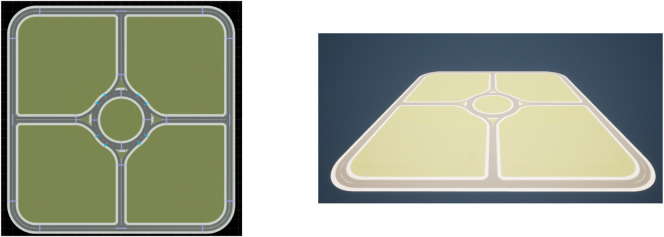
Map created in RoadRunner (left) and map converted for use in CARLA (right).

In order to generate the map of the area, we have to either create a map from scratch or obtain road data from OpenStreetMap and convert it into a format that can be used by CARLA and SUMO. The former option has been selected as there are compatibility issues between CARLA and SUMO when trying to implement a right-hand drive convention in the imported map. As a result, a left-hand drive format has been adopted, and the simulations are carried out in an ideal circular roundabout with identical approaches in terms of length and merge angle. To create the map, RoadRunner is used, which is a tool that can create road networks or import and edit maps from OpenStreetMap. In our case, the generated map spans a total area of 250 × 250 m^2^. The roads leading up to the roundabout have two lanes and are in all four cardinal directions. The roundabout has a single lane and a radius of 15 m, similar to the roundabout in Figure 1. The four arms have a length of 100 m each, with a perimeter road connecting the end points of each approach in a square with rounded corners, see Figure 7. Note that the perimeter road is not used in the simulations as it only serves to avoid any bugs within the simulation software.

The created map in RoadRunner is exported in filmbox (.fbx) and OpenDRIVE (.xodr) formats for use with CARLA and SUMO, respectively. The fbx map can be easily integrated into CARLA/Unreal by using the plugins that have been developed alongside CARLA specifically for this task. The resultant map can then be saved as a CARLA project file for later use. To integrate the map created in RoadRunner with SUMO, the .xodr file has to be converted into a .net.xml file. Conveniently, the developers of CARLA have also designed a tool to automate this process and allow for smooth integration.

### 6.2 EGO vehicle control in CARLA

Once the map is ready, the EGO vehicle can be added to the simulation environment. This is performed in CARLA as the vehicles spawned in SUMO cannot be finely controlled to implement advanced motion planning algorithms. The EGO vehicle starts from the north of the map, approaches the roundabout, and then drives through and exits to the south, where the journey endpoint is situated. The EGO vehicle is spawned as a normal vehicle in CARLA, but with its autopilot turned off. It moves by defining GPS coordinates along its trajectory every 50 ms. These coordinates are also referred to as waypoints, and they are spaced out by how much the motion planning algorithm decides to move the EGO vehicle every 50 ms. Apparently, the speed and acceleration of the EGO vehicle can be determined by the distance separation between successive waypoints. For example, waypoints positioned further away than from the previous waypoints emulate acceleration, whereas equidistant waypoints indicate a constant velocity. The waypoints can be customized on the fly, allowing the driving algorithm to dynamically react to other traffic objects.

### 6.3 Traffic object control in SUMO

In SUMO, it is straightforward to spawn a continuous stream of vehicles at one point with random destinations to different parts of the map. For each vehicle, the destination is (randomly) selected at the moment the vehicle is generated, thereby ruling out the possibility to drive around the ring several times. The characteristics of road traffic can be determined by modifying stream parameters such as the generation probability of vehicles that determines how likely it is to spawn a vehicle from a specific location every 1 s. The lower the probability, the less likely a vehicle will spawn. Since 1 s is too short an interval to eliminate the chance of overlapping vehicles, SUMO uses a parameter called ‘minGap’, *g* *_min_*, which is the minimum gap (in meters) between successive vehicles when spawning them into the environment. Note that the ‘minGap’ is measured from the rear of the leading vehicle to the bumper of its follower. In our simulations, we have set *g* *_min_* = 0.4 m, which might appear to be small; however, ‘minGap’ is used in another function to calculate the minimum headway distance at the time of spawning *h*_*d*_ (Krajzewicz et al., 2012):

$$h_{d}=g_{\min}+h_{\tau }v_{\mathrm{spawn}},$$ (3)

where *v*_*spawn*_ is the spawning speed of the vehicle and *h*_*τ*_ is the minimum desired time headway between successive vehicles. In our simulations, we have set *v*_*spawn*_ = 6.7 m/s (15 mph) and *h*_*τ*_ = 1 s, yielding that *h*_*d*_ ≈ 7 m is the minimum headway distance between successive vehicles at spawning.

The default control algorithm in SUMO consists of the routing algorithm and the car-following model. The routing model is an algorithm that selects a random or fixed path whenever a traffic object reaches at an intersection with two or more exits. In our case, all traffic objects have an equally random chance of exiting from one of the possible three exits at the roundabout, discounting the direction the traffic object is coming from. This will result in a uniform distribution of traffic objects exiting at each possible exit.

The default SUMO driving algorithm is the Krauss car-following model (Krauss, 1998). The Krauss model finds a safe longitudinal speed^4^, *v*_*safe*_ to avoid mutual crashes, taking into consideration several parameters such as the current speed of the vehicle, *v*(*t*), the leading vehicle speed, *v*_*l*_(*t*), the distance headway to the leading vehicle, the driver’s reaction time *T*_*r*_, and the maximum deceleration *γ* *_max_* allowed on a vehicle. Mathematically, one can write that at time *t*,

$$v_{\mathrm{safe}}\left(t\right)=v_{l}\left(t\right)+{\left(\frac{v_{l}\left(t\right)-v\left(t\right)}{2\gamma_{\max}}+T_{r}\right)}^{-1}\left(g\left(t\right)+g_{\mathrm{des}}\left(t\right)\right),$$ (4)

where *g* and *g*_*des*_ are the current and desired gaps, respectively, to the leading vehicle.

In some cases, *v*_*safe*_(*t*) may be computed to be higher than the speed limit *v*_*max*_ or the achievable speed at the next time step bounded by the maximum acceleration. Therefore, the minimum of these values must be selected as the target speed of the vehicle (Song et al., 2015),

$$v_{\mathrm{target}}\left(t\right)=\min\left\{v_{\mathrm{safe}}\left(t\right),v_{\max},v\left(t\right)+\gamma_{\max}\delta t\right\},$$ (5)

where (*v*(*t*) + *γ*_*max*_ *δt*) is the speed at the next time step, i.e., *δt* = 50 ms in our simulations, bounded by the maximum acceleration *γ*_*max*_ that has been set equal to *γ*_*nax*_ = 2 m/s^2^.

The speed limit can be manually set in SUMO for the traffic objects or in CARLA for the EGO vehicle. A speed limit of *v*_*max*_ = 13.4 m/s (or 30 mph) has been selected as this is the national speed limit in built-up areas in the U.K.^5^. Finally, it should be noted that the Krauss model is also used by the traffic objects generated in SUMO to react to the EGO vehicle controlled by CARLA. In this case, information must be exchanged through the CARLA–SUMO co-simulation bridge (a script is provided by CARLA developers) that allows data conversion and communication between the two simulation environments.

### 6.4 SUMO and CARLA integration

The main components of the co-simulation environment are the map and the Python scripts which run the specific functions via the Python API of CARLA. To run the co-simulation, the maps used between CARLA and SUMO must be in sync and generated from the same original map. The latter is created in RoadRunner and can be shared between the two simulators using different file formats, as detailed in Section 6.1. To run the Python scripts that execute the functions and control the road traffic environment, the Python API built with CARLA is used. In this API, there are scripts that allow seamless bridging between CARLA and SUMO. Since our simulations assume that the positions and intended paths of traffic objects (generated at SUMO) become available to the EGO vehicle (generated in CARLA), the bridge had to be slightly modified to also relay such information.

**FIGURE 8:**
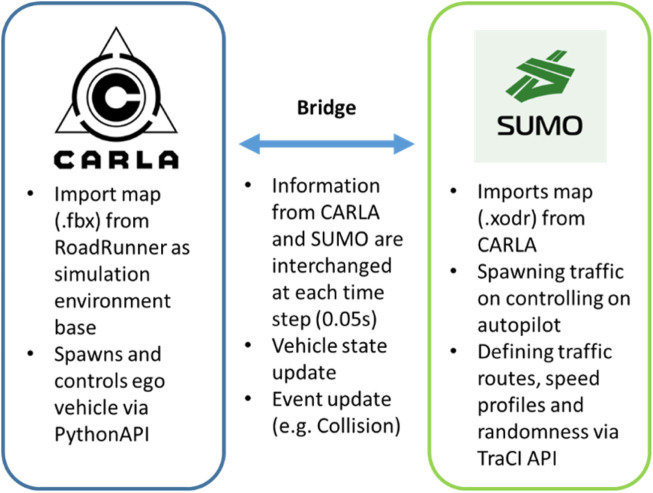
Co-simulation environment between CARLA and SUMO.

Figure 8 shows an example of information exchange. The 3D map and control of the EGO vehicle are performed on the CARLA side, whereas the 2D microscopic map and the traffic object control are performed on the SUMO side. The bridge seamlessly relays information between the two at a specified frequency synchronously ^6^. In this way, all data remain in sync with the time steps, and no data are lost or dropped.

### 6.5 Data collection workflow

Each simulation run starts with the EGO vehicle at 100 m from the north entrance of the roundabout and finishes once the EGO vehicle exits at the south approach. For each journey, the instantaneous positions, speeds, and accelerations of the EGO vehicle and of all traffic objects are stored in discrete time steps of 50 ms in .csv format. This allows for the simulation data to be analyzed and easily replayed. After all data are extracted, the trajectories of the EGO vehicle are first imported into MATLAB to calculate the driving efficiency and ride quality KPIs. To extract the safety KPIs, a program called Surrogate Safety Assessment Model (SSAM) is used to automatically process the simulated trajectory files and calculate the TTC and PET between the EGO vehicle and all traffic objects. The SSAM tool analyzes the trajectories of all vehicles within a simulation and extracts potential and/or actual collisions as conflicts using thresholds on the TTC and PET that can be adjusted by the user. In our case, the interaction between the EGO vehicle and a traffic object is classified as a potential conflict if their PET becomes less than 5 s or their TTC becomes less than 3 s at any point during their travel.

## 7 Evaluating the impact of collective perception

In this section, simulations are carried out to measure the effect of the additional information provided by the RSU through V2X communication on the driving efficiency, passenger comfort, and safety of an EGO vehicle traveling across an unsignalized urban roundabout. The baseline and the enhanced algorithms for motion planning presented in Section 3 and Section 4 are evaluated and compared against the KPIs for driving efficiency, ride quality, and safety determined in Section 5. A nominal flow rate of 8–16 vehicles/minute has been measured at roundabouts in central Europe (Ambros et al., 2016), which is used as a benchmark for parameterizing road traffic in our simulations. We consider only symmetric traffic by assigning the same spawning probability for vehicles in SUMO at each arm of the roundabout. Future work is required to measure the traffic densities at the four arms using infrastructure sensors (deployed in the junction but not included in the description of Section 2) and study the asymmetric traffic case.

### 7.1 Simulation scenario setup

To model urban environments with different road traffic conditions, we vary the spawning probability of vehicles in SUMO. A new vehicle is generated every second at the west, east, and south arms of the roundabout with probability $\left\{3,6,9,12,15,20\right\}\;\%$ and at 100 m distance from the corresponding entrance. A spawning probability of 3% is associated with a flow rate of 0.03 × 3 × 60 = 5.4 vehicles/minute, where the factor × 3 indicates symmetric traffic at the three approaches of the roundabout. Note that a spawning probability of 20% may correspond to a flow rate slightly less than 36 vehicles/minute because of the headway constraints between successive vehicles discussed in Eq. 3. Initially, we do not generate any traffic object at the north arm to avoid the EGO vehicle from being caught up or slowed down by traffic in front of it while approaching the roundabout, as some of the collected KPIs would be affected and dictated by the road traffic in front instead of the EGO vehicle and the infrastructure. Nevertheless, the KPIs will also be evaluated with random traffic spawned at all arms of the roundabout later in this section. For each spawning probability, 1,000 journeys for the EGO vehicle are simulated.

### 7.2 Results and discussion

**FIGURE 9:**
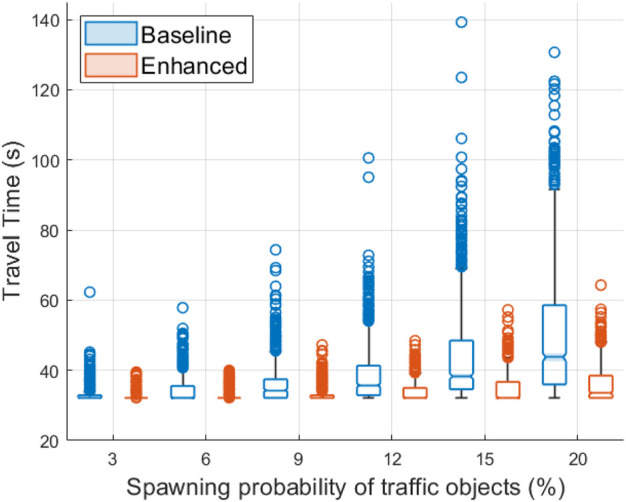
Travel time box plot for the EGO vehicle using the baseline and the enhanced algorithms. Higher spawning probabilities of traffic objects are associated with increasing road traffic densities.

**FIGURE 10:**
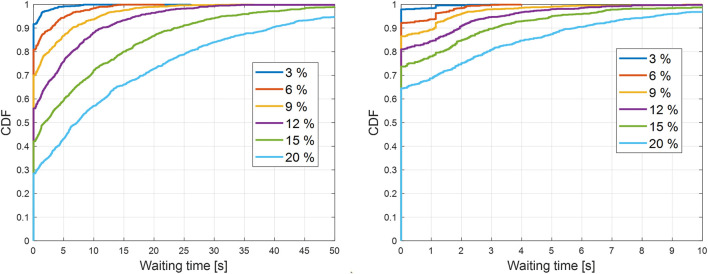
Empirical cumulative distribution function (CDF) of stationary waiting time for the EGO vehicle at the north entrance of the roundabout for the baseline (left) and the enhanced (right) algorithm. The clock to measure the waiting time starts ticking when the velocity of the EGO vehicle is less than 0.45 m/s (or 1 mph). Higher spawning probabilities of road traffic in the legend are associated with increasing road traffic densities.

**FIGURE 11:**
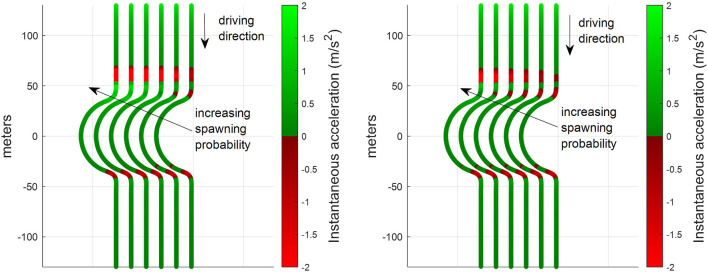
Color-coded instantaneous acceleration of the EGO vehicle along its route for different spawning probabilities $\left\{3,6,9,12,15,20\right\}\%$ using the baseline (left) and the enhanced (right) algorithms. The acceleration for every point along the route is calculated as the average over 1,000 journeys. Higher spawning probabilities of road traffic in SUMO are associated with increasing road traffic densities. Since an ideal circular roundabout is used in our simulations, as discussed in Section 6.1, we could not illustrate the acceleration profile of the EGO vehicle on the top of Google Maps depicted in Figure 1.

**FIGURE 12:**
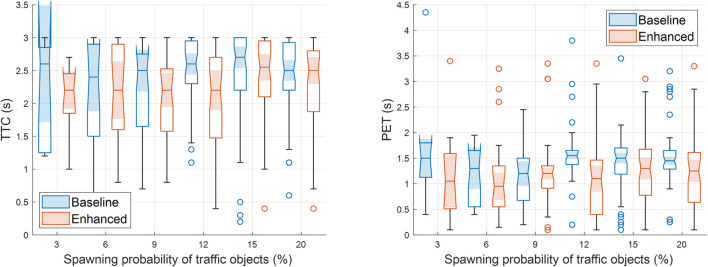
Box plots for the time-to-collision (left) and the post-encroachment time (right) with the baseline and the enhanced algorithms. Higher spawning probabilities of traffic objects are associated with increasing road traffic densities in SUMO. Recall that the maximum PET and TTC for identifying potential conflicts in SSAM are set equal to 5 s and 3 s, respectively.

The simulation results depicted in Figure 9 and Figure 10 confirm that cooperative perception can significantly improve the driving efficiency of the EGO vehicle both in terms of journey completion time and waiting time at the entrance of the roundabout. The EGO vehicle becomes aware of the intended trajectories of other vehicles, and because of that, it is less likely to come to a complete stop at the entrance. At the highest simulated flow rate, the EGO vehicle stops with probabilities of 70% using the baseline algorithm and only 35% using the enhanced algorithm. Cooperative perception also enhances the journey comfort, especially near the entrance, as can be seen in the acceleration plots in Figure 11. Over there, the baseline algorithm abruptly decelerates the EGO vehicle even at moderate traffic densities, e.g., for a spawning probability equal to 6%, before accelerating again. With the enhanced algorithm, the acceleration profile becomes much smoother near the entrance, and thus the journey is more comfortable. Since the driving efficiency and comfort improve, it is expected that the EGO vehicle will either experience smaller margins than other vehicles or make riskier maneuvers. Hence, as an educated guess, the safety proximity metrics are expected to be slightly worse or similar when comparing the enhanced to the baseline algorithm. It can be observed in Figure 12 that the TTC and PET for the two algorithms are not statistically different. This can be seen by noticing that the shaded notched areas of the box plots for the same traffic flow rate significantly overlap. Therefore, it is safe to deduce that despite the driving efficiency and ride quality gains, there are no adverse effects on the TTC and PET in this specific case.

**FIGURE 13:**
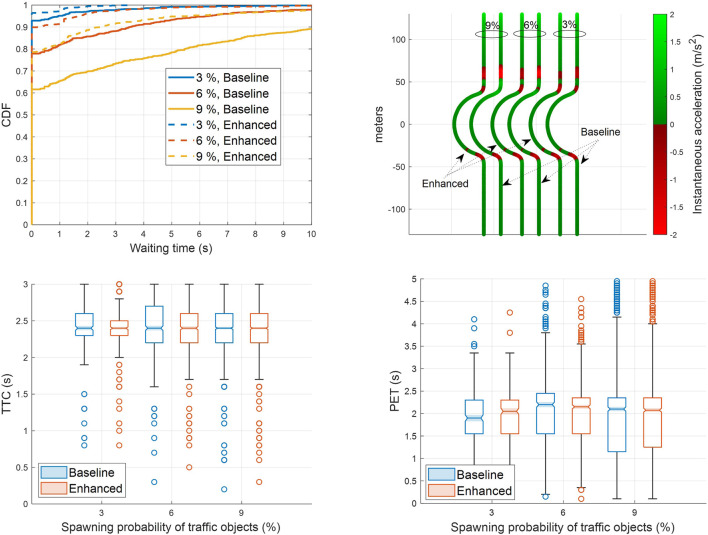
Performance of the baseline and enhanced algorithms in terms of waiting time (top-left), ride quality (top-right), time-to-collision (bottom-left), and post-encroachment time (bottom-right) for spawning probabilities of road traffic $\left\{3,6,9\right\}\%$ at all approaches of the roundabout, i.e., road traffic also generated in the northbound/EGO vehicle’s lane.

We conclude this section by comparing the two algorithms when random road traffic is generated also at the north arm of the roundabout. The performance evaluation is summarized in Figure 13 for three different traffic flow rates. For spawning probability 9%, which corresponds to approximately 0.09 × 4 × 60 = 21.6 vehicles/min, the baseline algorithm stops the EGO vehicle at the entrance with a probability of 40%, while the enhanced algorithm does that only for 20% of the simulated journeys. The enhanced algorithm also improves passenger comfort, inducing less deceleration and acceleration at the entrance of the roundabout. Finally, the TTC and the PET for the two algorithms remain approximately the same for the same flow rate.

## 8 Conclusion and future work

In order to realize the vision of level 4 autonomy, future vehicles must be able to acquire a faithful representation of the surrounding environment and road traffic. Solely relying on onboard perception to achieve that, such as human drivers rely on their decision-making to drive from point A to point B, will not be always sufficient. In this paper, we have considered such a case where an EGO vehicle approaches an unsignalized roundabout and cannot perceive vehicles approaching the roundabout from the other three entrances due to occlusions. In this scenario, we have acquired simulation evidence that a roadside infrastructure with edge computing capabilities and wireless connectivity, broadcasting the intended trajectories of road users, can significantly reduce the waiting time at the entrance of the roundabout for a connected EGO vehicle. Even though a rudimentary motion prediction module is adopted, the EGO vehicle can successfully avoid hazards. In the absence of hazards, the EGO vehicle does not stop at the entrance but continues its journey, saving time and enhancing ride quality, as it avoids unnecessary decelerations and jerky maneuvers near the entrance, without compromising safety. In the future, it is important to consolidate the outcomes of our study by modeling imperfect object detection and V2X communication, non-zero processing delays, and mixed-traffic conditions, where only some of the vehicles can share their intentions with the roadside unit. Furthermore, it is desirable to replace the PID controller of the EGO vehicle with a sophisticated car-following model because a PID controller cannot manage all traffic flow situations. As the penetration rate of connected and automated vehicles (CAVs) will increase, centralized coordination or distributed cooperative solutions among CAVs can also assist in decision-making, opening further directions for future work. We believe that the promising results obtained in this article can spark more research on CAVs navigating complex junctions such as A-roads, T-intersections, and motorway merges.

## Data Availability

All data are provided in full in the results section of this page.

## Footnotes

1
https://www.gov.uk/government/news/self-driving-revolution-to-boost-economy-and-improve-road-safety

2
https://carla.org/2020/09/25/release-0.9.10/

3
https://carla.readthedocs.io/en/latest/bp_library/

4 As SUMO always assumes that vehicles will stick to each lane, respectively, an explicit lateral control model is not relevant here.

5 U.K. Government, “National Speed Limits, United Kingdom”, https://www.gov.uk/speed-limits

6 CARLA Simulation time-step and synchrony, https://carla.readthedocs.io/en/latest/adv_synchrony_timestep/
